# Pharmacokinetics and Efficacy of the FimH Inhibitor Sibofimloc in Preventing Necrotizing Enterocolitis in Preterm Piglets

**DOI:** 10.64898/2026.10.12.758546

**Published:** 2026-10-13

**Authors:** Valeria Melendez Hebib, Brooklynn Earls, Barbara Stoll, Caitlin Vonderohe, Gregory Guthrie, Henry Joseph Haiser, Martin Paton, Suresh K. Balani, Matthias Hesse, Douglas Burrin

## Abstract

Necrotizing enterocolitis (NEC) is a disease in preterm infants associated with an increased abundance of Enterobacteriaceae. FimH, a mannose-binding adhesin on Enterobacteriaceae type 1 fimbriae, mediates mucosal attachment and TLR4-dependent inflammatory cascades. We evaluated the safety, pharmacokinetics, and efficacy of an oral FimH inhibitor (Sibofimloc) to reduce Enterobacteriaceae colonization and prevent NEC in preterm piglets. We performed a 9-d dose-escalation safety study (10, 25, 50 mg/kg BID) assessing absorption and tolerability and a 10-d randomized efficacy study. Piglets in the efficacy study received an infant formula diet with or without oral sibofimloc (40 mg/kg BID), or donor human milk (DHM) as a healthy control. NEC incidence, tissue histopathology, and gut microbiota composition via metagenomic whole-genome sequencing (WGS) were evaluated. Systemic exposure data reveal that sibofimloc was dose-dependently absorbed, while plasma levels decreased from postnatal day 2 to day 8, indicating reduced drug absorption with maturation of the intestinal barrier. Treatment with sibofimloc did not reduce NEC incidence (100% in sibofimloc in formula controls) or severity, whereas DHM-fed piglets experienced 0% NEC. Sibofimloc-treated animals exhibited significantly reduced crypt depths, demonstrating heightened mucosal injury and impaired regenerative capacity. WGS revealed that sibofimloc-treated animals maintained a gut luminal microbiota composition similar to untreated formula controls, dominated by *Clostridium perfringens* ( *C. perfringens* ). Selective targeting of FimH-expressing Enterobacteriaceae was insufficient to prevent NEC or alter the gut microbiota in this preterm piglet model, underscoring the complexity of NEC-associated microbial communities and suggesting that additional microbial targets and other colonization mechanisms may contribute to disease development.

